# Complexity Changes in the US and China’s Stock Markets: Differences, Causes, and Wider Social Implications

**DOI:** 10.3390/e22010075

**Published:** 2020-01-06

**Authors:** Jianbo Gao, Yunfei Hou, Fangli Fan, Feiyan Liu

**Affiliations:** 1 Center for Geodata and Analysis, Faculty of Geographical Science, Beijing Normal University, Beijing 100875, China; jbgao.pmb@gmail.com (J.G.); fanfang_li@163.com (F.F.); 2 Institute of Automation, Chinese Academy of Sciences, Beijing 100190, China; 3 International College, Guangxi University, Nanning 530004, China; 4 School of Economics and Management, Wuhan University, Wuhan 430072, China; 5 CityDO, Hangzhou 310000, China

**Keywords:** EMH, Lempel–Ziv complexity, permutation entropy, Hurst parameter, the US and China’s stock market

## Abstract

How different are the emerging and the well-developed stock markets in terms of efficiency? To gain insights into this question, we compared an important emerging market, the Chinese stock market, and the largest and the most developed market, the US stock market. Specifically, we computed the Lempel–Ziv complexity (LZ) and the permutation entropy (PE) from two composite stock indices, the Shanghai stock exchange composite index (SSE) and the Dow Jones industrial average (DJIA), for both low-frequency (daily) and high-frequency (minute-to-minute)stock index data. We found that the US market is basically fully random and consistent with efficient market hypothesis (EMH), irrespective of whether low- or high-frequency stock index data are used. The Chinese market is also largely consistent with the EMH when low-frequency data are used. However, a completely different picture emerges when the high-frequency stock index data are used, irrespective of whether the LZ or PE is computed. In particular, the PE decreases substantially in two significant time windows, each encompassing a rapid market rise and then a few gigantic stock crashes. To gain further insights into the causes of the difference in the complexity changes in the two markets, we computed the Hurst parameter *H* from the high-frequency stock index data of the two markets and examined their temporal variations. We found that in stark contrast with the US market, whose *H* is always close to 1/2, which indicates fully random behavior, for the Chinese market, *H* deviates from 1/2 significantly for time scales up to about 10 min within a day, and varies systemically similar to the PE for time scales from about 10 min to a day. This opens the door for large-scale collective behavior to occur in the Chinese market, including herding behavior and large-scale manipulation as a result of inside information.

## 1. Introduction

It is generally thought that the level of development of a capital market of a country is closely related to the degree of its economic development. A healthy capital market can provide an effective platform for financing the enterprise of the country. To realize this goal, stock prices in the market have to fluctuate randomly [1], so that wealth will not be drawn out of the market by simple exploitation of the systemic patterns in the market. When the prices of a market deviate from being completely random, the behaviors of the market are considered to be inconsistent with what efficient market hypothesis (EMH) [2,3,4] stipulates.

There has been much effort to empirically test the validity of EMH in various markets. Major approaches include using traditional statistical methods such as autocorrelation or cross-correlation [5,6,7], variance ratio [8,9,10,11], the state space model [12], as well as metrics from complexity science such as Lempel–Ziv complexity (LZ) [13,14,15,16,17,18,19], permutation entropy (PE) [20,21,22,23,24,25,26,27,28,29,30,31,32,33], and Hurst parameter and multifractal measures [11,34,35,36,37,38,39,40,41,42,43,44,45,46,47,48,49,50,51,52]. The approaches from complexity science are especially appealing as they are fundamentally different from machine-learning-based black-box approaches. In particular, complexity science is rich in concepts, theories, and models, and thus can not only offer innovative solutions in vastly different scenarios, but also tremendously help with problem formulation. It is especially interesting to note that complexity science has been successfully applied to study many important problems in humanities, including disasters, crime, terrorism, wars, and epidemics [53,54].

As most published studies use low-frequency daily stock index data to test the validity of EMH in various markets [25,26,27,28], they only point out whether a market was efficient or not in a certain time span (such as a few years), but do not shed much light on the temporal variation of the market efficiency, nor help to characterize the difference in the efficiency between different markets. While the finding that developed markets are more consistent with the EMH than the emerging ones [26] is very appealing, the reported PE value for emerging markets is actually very close to a scenario of complete randomness, which is 1. The few studies that used high-frequency data were more stimulating [18,20] as it was found that minute-to-minute price data were slightly (but statistically significantly) more predictable than daily prices [18], and on very short time scales, stock data demonstrated microbehaviors that were not fully random. Unfortunately, the high-frequency data used in those studies only covered a short time span, and thus were not quite viable for examining the dynamical changes in market efficiency. Fortunately, this shortcoming can be readily overcome by working with high-frequency data collected over a long time span, as shown by a recent analysis of the Chinese stock market using minute-to-minute stock price data, where it was found that the values of PE can be significantly smaller than 1 [29]. In this paper, we examine whether similar results can be obtained with developed markets using high-frequency data, and whether the difference between emerging and developed markets can be better captured by high-frequency data.

To answer the above questions, and specifically to gain insights into the temporal variations in efficiency and complexity between emerging and developed stock markets, we chose to compare two important markets, the US market, which is the largest, the best developed, and the most important market, and the Chinese market, which is the most observed emerging market in the world. We will examine both low-frequency (daily) and high-frequency (minute-to-minute) stock index data by computing the Lempel–Ziv complexity (LZ), the permutation entropy (PE), and the Hurst parameter, and study how the two markets differ at different time scales and in different time windows.

The remainder of the paper is organized as follows. In Section 2, we describe the data used in this study, the algorithms of LZ, PE, and the adaptive fractal analysis (AFA) for computing the Hurst parameter *H*. In Section 3, we compute the LZ and the PE of the US and Chinese stock markets using both the low- and high-frequency data, compare their complexity changes, and finally compute the Hurst parameter of these two markets using high-frequency data to further clarify the cause of complexity changes. In Section 4, we make the concluding remarks.

## 2. Data and Methods

### 2.1. Data

We analyzed both the daily and minute-to-minute composite indices of the Shanghai stock market (SSE), China, from 2 January 2003 to 8 August 2016, and the Dow Jones industrial average (DJIA) of the US market from 2 January 2003 to 31 December 2014. In China, generally, the trading time in a trading day is from 9:30 to 11:30 in the morning and 13:00 to 15:00 in the afternoon, while in the US, the trading time is from 9:30 to 16:00. Thus, there are 240 min by minute data points for the SSE and 390 points for the DJIA on each trading day, Monday through Friday.

Concretely, we analyzed the logarithmic returns of the composite indices of SSE and DJIA,

(1) $$R_{t}=\ln\left(P_{t}\right)-\ln\left(P_{t-1}\right).$$

### 2.2. Methods

#### 2.2.1. Lempel–Ziv (LZ) Complexity

The LZ complexity [55] and its derivatives are closely related to the Kolmogorov complexity [56,57] and the Shannon entropy. They can be easily computed and have found wide applications in characterizing the randomness of complex data.

To compute the LZ complexity, a numerical sequence has to first be transformed into a symbolic sequence. The most popular approach is to convert the signal into a 0–1 sequence by comparing the signal with a threshold value $S_{d}$ [14]. That is, whenever the signal is larger than $S_{d}$, one maps the signal to 1; otherwise, one maps it to 0. One good choice of $S_{d}$ is the median of the signal [15]. When multiple threshold values are used, one may map the numerical sequence to a multisymbol sequence. Note that if the original numerical sequence is a nonstationary random walk-type process, one should analyze the stationary difference data instead of the original nonstationary data.

After the symbolic sequence is obtained, it can then be parsed to yield distinct words, and the words can be encoded. Let L(n) denote the length of the encoded sequence for those words. The LZ complexity can be computed as

(2) $$C_{LZ}=\frac{L(n)}{n}.$$

Note, this is very much in the spirit of the Kolmogorov complexity [56,57].

There exist many different methods for performing parsing. One popular scheme was proposed by the original authors of the LZ complexity [55]. Another attractive method is proposed by Cover and Thomas [58]. Let c(n) denote the number of words in the parsing of the source sequence by the second scheme. For each word, we can use $\log_{2}c\left(n\right)$ bits to describe the location of the prefix to the word and one bit to describe the last bit. Then, the total length of the encoded sequence is $L\left(n\right)=c\left(n\right)[\log_{2}c\left(n\right)+1]$. Equation (2) then becomes

(3) $$C_{LZ}=c\left(n\right)\left[\log_{2}c\left(n\right)+1\right]/n.$$

When *n* is very large, $c\left(n\right)\leq n/\log_{2}n$ [55,58]. Dropping terms much smaller than $\log_{2}n$, we can replace $\log_{2}c\left(n\right)$ in Equation (3) by $\log_{2}n$ and obtain

(4) $$C_{LZ}=\frac{c(n)}{n/\log_{2}n}.$$

This is the functional form for the definition of the commonly used LZ complexity. Unfortunately, $C_{LZ}$ depends on the sequence length. Rapp et al. [59] were among the first to consider normalizing the LZ complexity to make it independent of the sequence length by computational means. This issue was reconsidered by Hu et al. [60] using an analytic approach.

#### 2.2.2. Permutation Entropy (PE)

PE is introduced in [21] as a convenient means of analyzing a time series. It may be considered as a measure from chaos theory, since embedding vectors are used in the analysis. Using the notations of [22], it can be described as follows.

For a given but otherwise arbitrary *i*, the *m* number of the real values of $X_{i}=\left[x\left(i\right),x\left(i+L\right),\ldots ,x\left(i+\left(m-1\right)L\right)\right]$ are sorted in ascending order, $[x\left(i+\left(j_{1}-1\right)L\right)\leq x\left(i+\left(j_{2}-1\right)L\right)\leq \cdots \leq x\left(i+\left(j_{m}-1\right)L\right]$. When an equality occurs, e.g., $x\left(i+\left(j_{i1}-1\right)L\right)=x\left(i+\left(j_{i2}-1\right)L\right)$, the quantities *x* are ordered according to the values of their corresponding *j*’s; that is, if $j_{i1}<j_{i2}$, then we write $x\left(i+\left(j_{i1}-1\right)L\right)\leq x\left(i+\left(j_{i2}-1\right)L\right)$. Therefore, the vector $X_{i}$ is mapped onto a sequence of numbers, $\left(j_{1},j_{2},\ldots ,j_{m}\right)$, which is one of the m! permutations of *m* distinct symbols (1,2,…,m). When each such permutation is considered as a symbol, the reconstructed trajectory in the *m*-dimensional space is represented by a symbol sequence. Let the probability for the K≤m! distinct symbols be $P_{1},P_{2},\ldots ,P_{K}$. Then, PE, denoted by $E_{p}$, for the time series {x(i),i=1,2,…} is defined as

(5) $$E_{p}\left(m\right)=-\sum_{j=1}^{K}P_{j}\ln P_{j}.$$

The maximum of $E_{P}\left(m\right)$ is ln(m!) when $P_{j}=1/\left(m!\right)$. It is convenient to work with

(6) $$0\leq E_{p}=E_{p}\left(m\right)/\ln\left(m!\right)\leq 1.$$

Thus, $E_{p}$ gives a measure of the departure of the time series under study from a completely random one: the smaller the value of $E_{p}$ is, the more structure the time series has.

To detect interesting dynamical changes in a time series, one can partition a time series into overlapping or nonoverlapping segments of short length, compute the PE from each segment, and examine how the PE changes with the segments [22]. Here, we apply this approach to compute the PE from the minute-to-minute logarithmic yields of the composite indices of SSE and DJIA on each trading day, then check how the PE varies with time.

In this paper, for the daily stock index data of both USA and China, we employ the same segmentation and choose m=5 and L=1. For the intraday stock index data, since on each day, the data for the Chinese market is shorter than for the USA, we choose m=4, L=1 for SSE and m=5, L=1 for DJIA, respectively. Note that the time delay *L* was always chosen to be 1; this is based on the reasoning that stock data are basically random. As explained in [36], such a choice is not only sufficient, but optimal. The selection of *m* is basically constrained by the length of each sub-dataset under computation—if the data segment is short, then *m* cannot be too big. To better cope with the randomness in the data, *m* should not be too small either. The fact that the embedding window (m−1)L is often larger than 1 (and as a result of the patterns in the dataset) makes it possible to quantify the correlations in the data to some degree with the PE. This will be discussed in more depth later in the paper.

#### 2.2.3. Adaptive Fractal Analysis (AFA)

Consider a $1/f^{2H+1}$ process, where 0<H<1. Such processes are normally called nonstationary random-walk processes. Its differentiation, denoted as $X=\{x_{t}:t=0,1,2,\ldots \}$, is a covariance stationary stochastic process, with mean μ, variance $\sigma^{2}$, and autocorrelation function r(w) have the form [35]

(7) $$r\left(w\right)∼w^{2H-2},\;\;as\;\;w\to \infty .$$

When 1/2<H<1, $\sum_{w}r\left(w\right)=\infty$, leading to long-range temporal correlation. The process *X* has a PSD of $1/f^{2H-1}$. A 1/f process cannot be aptly modeled by a Markov process or an ARIMA model [61] since the PSD of those processes are distinctly different from 1/f. To adequately model a 1/f process, a fractional order process has to be used. A well-known process of this class is the fractional Brownian motion model [34].

There are many excellent methods to estimate the Hurst parameter [36]. One of the most popular methods for estimating the Hurst parameter *H* is detrended fluctuation analysis (DFA) [62]. This involves constructing a random walk process

(8) $$u\left(n\right)=\sum_{k=1}^{n}\left(x_{k}-\bar{x}\right),\;\;i=1,2,\ldots ,n,$$

where $\bar{x}$ is the mean of the series x(i),i=1,2,…, dividing the constructed random walk process into nonoverlapping segments, determining the best linear or polynomial fits in each segment as the local trends, getting the variance of the differences between the random walk process and the local trends, and averaging them over all the segments. Clearly, DFA may involve discontinuities at the boundaries of adjacent segments. Such discontinuities could be detrimental when the data contain trends [63], or nonstationarity [64] or nonlinear oscillatory components such as signs of rhythmic activity [65,66]. Fortunately, this shortcoming can be readily overcome using a method called adaptive fractal analysis (AFA) [38,39]. AFA is based on a nonlinear adaptive multiscale decomposition, which starts by partitioning a time series into segments of length w=2n+1, where neighboring segments overlap by n+1 points. Each segment is then fitted with the best polynomial of order *M*. We denote the fitted polynomials for the *i*-th and (i+1)-th segments by $y^{\left(i\right)}\left(l_{1}\right)$ and $y^{\left(i+1\right)}\left(l_{2}\right)$, respectively, where $l_{1},l_{2}=1,\ldots ,2n+1$. We then define the fitting for the overlapped region as

(9) $$y^{\left(c\right)}\left(l\right)=w_{1}y^{\left(i\right)}\left(l+n\right)+w_{2}y^{\left(i+1\right)}\left(l\right),\;\;l=1,2,\ldots ,n+1,$$

where $w_{1}=\left(1-\frac{l-1}{n}\right)$ and $w_{2}=\frac{l-1}{n}$ can be written as $\left(1-d_{j}/n\right)$ for j=1,2, and where $d_{j}$ denotes the distances between the point and the centers of $y^{\left(i\right)}$ and $y^{\left(i+1\right)}$, respectively. This means that the weights decrease linearly with the distance between the point and the center of the segment. Such a weighting ensures symmetry and effectively eliminates any jumps or discontinuities around the boundaries of neighboring segments, and therefore can maximally suppress the effect of complex nonlinear trends on the scaling analysis.

With the above procedure, AFA can be readily described. For an arbitrary window size *w*, we determine, for the random walk process u(i), a global trend v(i),i=1,2,…,N, where *N* is the length of the walk. The residual, u(i)−v(i), characterizes fluctuations around the global trend, and its variance yields the Hurst parameter *H* according to

(10) $$F\left(w\right)={\left[\frac{1}{N}\sum_{i=1}^{N}{\left(u\left(i\right)-v\left(i\right)\right)}^{2}\right]}^{1/2}∼w^{H}.$$

## 3. Results

### 3.1. Detecting Complexity Changes by LZ and PE Using Low-Frequency Data

**Figure 1:**
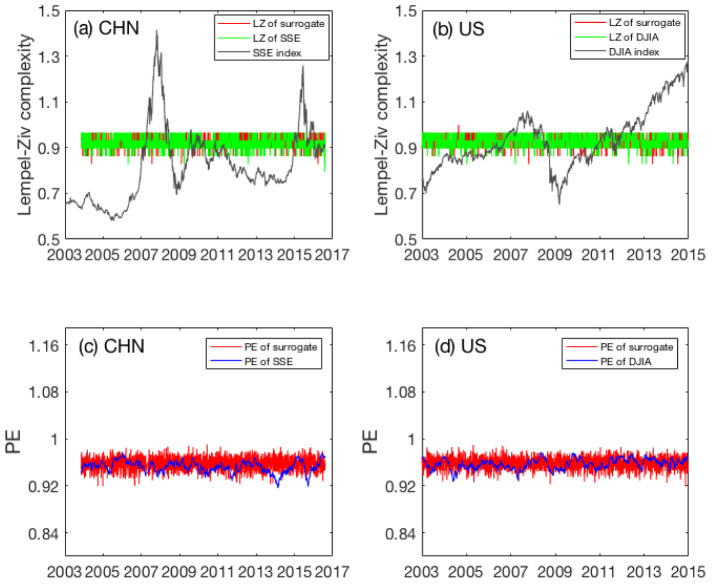
Temporal variations of Lempel–Ziv complexity (LZ) (the green curves) and permutation entropy (PE) (the blue curves) for the Chinese (**a**,**c**) and the US (**b**,**d**) markets. The curves were computed from daily stock data, using a moving window of size 200 days, where adjacent windows overlap by 199 days. The LZ and PE of the shuffled data for the Shanghai stock exchange composite index (SSE) and Dow Jones industrial average (DJIA) were also plotted as red curves.

We examined whether low-frequency stock data can be used for detecting the complexity changes in the Chinese and the US stock markets by computing the temporal variations of LZ and PE using daily stock index data. The results for LZ and PE are shown as the green and blue curves in Figure 1a–d, respectively. The curves were computed from daily stock data, using a moving window of size 200 days, where adjacent windows overlap by 199 days. LZ and PE were also computed from the shuffled data without any correlations. They are shown in the plots as the red curves. It is observed that the red curves are essentially indistinguishable from the green and blue curves computed from the daily stock data in these two stock markets. Therefore, the daily SSE and DJIA data are essentially random. Consequentially, the behavior of both markets is basically consistent with the EMH, when time scales of 1 day and longer are concerned.

The above result has two interesting implications: (1) daily low-frequency stock data may not be viable for detecting complexity changes in SSE and DJIA; (2) LZ or PE may not be capable of characterizing complexity changes in the two stock markets. In the next subsection, to find out which implication is more relevant, we examine the variations of LZ and PE using high-frequency stock data.

### 3.2. Detecting Complexity Changes by LZ and PE Using High-Frequency Data

**Figure 2:**
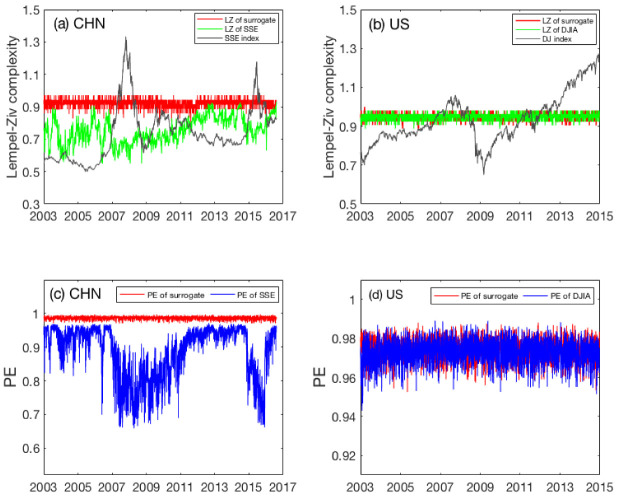
Temporal variations of LZ (the green curves) and PE (the blue curves) for the Chinese (**a**,**c**) and the US (**b**,**d**) stock markets. The computation was performed day by day using minute-to-minute data. As benchmark, the LZ and PE of the shuffled data for SSE and DJIA are also shown as red curves.

With the minute-to-minute high-frequency stock data, we are able to compute LZ and PE on each day and examine their temporal variations. The results for LZ and PE are summarized in Figure 2a–d, respectively. As benchmark, LZ and PE were also computed from the shuffled data without any correlations and plotted as the red curves in the plots. For the US market, what we observe from Figure 2b,d is basically the same as what is revealed by the low-frequency daily data shown in Figure 1b,d: the market is essentially random. However, a completely different picture emerges for the Chinese market. Concretely, we observe from Figure 2a that LZ for the Chinese stock market fluctuates wildly during the entire time span from 2003 to 2016 and is significantly smaller than the LZ for the fully random shuffled data. This plainly suggests that the Chinese stock market usually violates the EMH. The plot of PE is even more interesting. As shown in Figure 2c, PE for the Chinese stock market not only fluctuates wildly during the entire time span, but also decreases substantially in two significant time windows, one being from mid-2006 to mid-2011, the other from the end of 2014 to 2016. Each period encompasses a rapid market rise (bull market) and then a few gigantic stock crashes. As the first period also encompasses the global financial crisis, it is much longer than the second period. While both PE and LZ have indicated unambiguously that the Chinese stock market is highly inconsistent with the EMH on time scales shorter than a day, we have to conclude that PE offers a better means of characterizing the complexity changes in the Chinese stock market than LZ when high-frequency stock index data are used.

### 3.3. Cause of Complexity Changes: Long-Range Correlation

**Figure 3:**
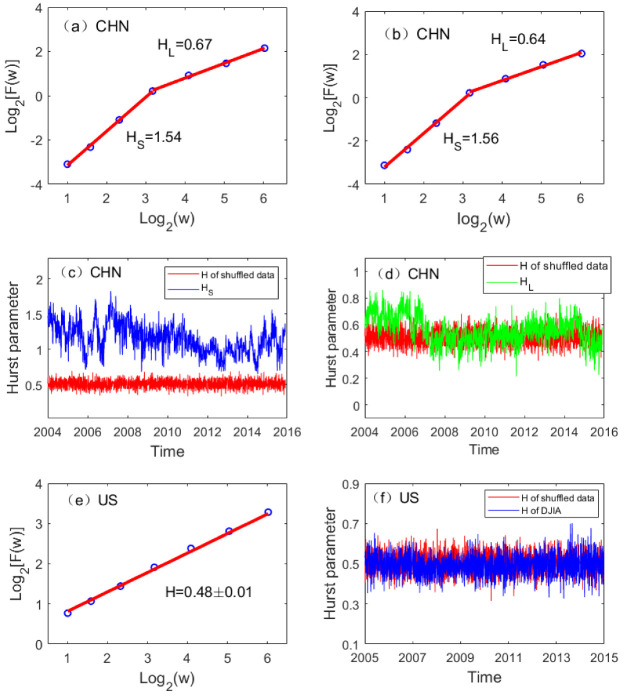
Adaptive fractal analysis assessing the long-range correlations in the Chinese and the US stock markets: (**a**,**b**,**e**) $\log_{2}F\left(w\right)$ vs. $\log_{2}w$ for arbitrarily chosen days in China and the USA, which exhibits two scaling regimes (with slopes denoted as $H_{s}$ and $H_{L}$) for China’s market, and a simple scaling similar to a Brownian motion for the US market; (**c**,**d**) temporal variations of $H_{s}$ and $H_{L}$ for China, where $H_{s}$ differs from 1/2 significantly, while $H_{L}$ shows systematic variations consistent with the timing of the two strong bull markets, occurring in the middle of 2006 and the end of 2014; H≈0.5 for the shuffled data is shown as red curves in the plots; (**f**) temporal variation of *H* for the USA, the value of which is close to 1/2 and so indistinguishable from that of the shuffled data.

To gain further insights into the mechanism for the huge difference between the complexity changes in the Chinese and the US stock markets, we carried out AFA of the high-frequency minute-to-minute stock data on each day for the two markets and examined the temporal variation of the Hurst parameter. Two examples of typical fractal scaling $\log_{2}F\left(w\right)$ vs. $\log_{2}w$ curves for the Chinese market are shown in Figure 3a,b, where we observe two distinct scaling regimes, one is from 1 min to about 10 min, with an $H_{S}$ larger than 1. The other is for time scales above 10 minutes, with an $1/2<H_{L}<1$. In contrast, the US stock data only exhibits a single scaling, with an *H* very close to 1/2, as shown in Figure 3e. The variations of $H_{S}$ and $H_{L}$ with time for the Chinese market and the *H* for the US market are shown in Figure 3c,d,f, together with a curve of *H* (designated as red) for the fully random shuffled data. We observe that the behavior of the US market is consistent with the EMH, and is fully consistent with what was revealed by LZ and PE. However, the behavior of the Chinese market is totally different; while $H_{S}$ is often larger than 1 and fluctuates widely with time, $H_{L}$ also varies systematically. In fact, on the basis of the variations of $H_{L}$, three periods can be identified. One is from 2004 to mid-2006, the second is from mid-2006 to the end of 2014, the third is from the end of 2014 to 2016. During each period, $H_{L}$ steadily increases. As mid-2006 and the end of 2014 coincide with the two strongest bull markets in China, the variation of $H_{L}$ suggests that the decrease in $H_{L}$ coincides with the occurrence of the bull markets. Not only so, the mean level of $H_{L}$ during those two bull market periods is smaller than 0.5, highlighting the persistence of the two bull markets—the antipersistent correlations characterized by $H_{L}<0.5$ means that any deviations from a bull market are soon stabilized. Indeed, both bull markets were sustained for about half year because of favorable governmental policies, even though basic economic conditions in those two periods were not very different from other periods.

### 3.4. Correlation between LZ, PE, and H for SSE and DJIA

**Figure 4:**
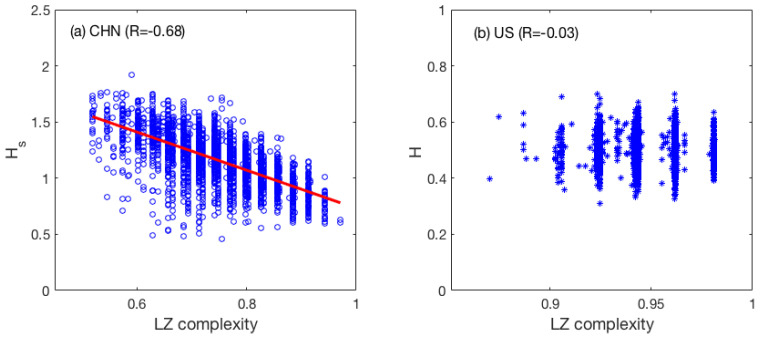
Scatter plots of H and LZ for the two stock markets: (**a**) the Chinese market (**b**) the US market.

To examine whether correlations exist between LZ and the Hurst parameter, we can simply plot the scatter plots between LZ and *H*. As can be seen from Figure 4, indeed correlations exist between $H_{S}$ and LZ for the Chinese market. The correlation coefficients between *H* and LZ for SSE and DJIA are 0.68 and −0.03, respectively. The lack of any correlation between H and LZ for the US market is as expected, since *H* corresponds to the fully random case and essentially does not change with time (and thus cannot be expected to be correlated with any variable). What is surprising is that for the Chinese market, a strong negative correlation does exist between LZ and the short time scale *H*. This is consistent with our finding that the Chinese market has some nonrandom structural properties on time scales shorter than 10 min.

**Figure 5:**
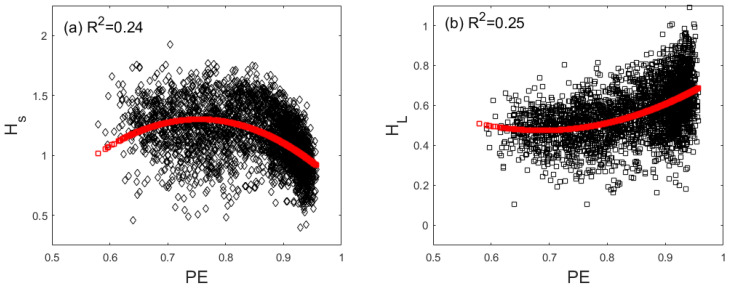
Scatter plots of H and PE for the Chinese market. (**a**) $H_{S}$ vs. PE, (**b**) $H_{L}$ vs. PE.

The correlations between PE and *H* can also be examined by plotting the scatter plots. As shown in Figure 5a,b, we observe that PE has fairly strong nonlinear correlations with *H* on both short and long time scales for the Chinese market. In particular, the largely positive and weakly nonlinear correlation between $H_{L}$ and PE suggests that the variation of $H_{L}$ with time would be similar to the variation of PE with time. Indeed, this is clearly shown in Figure 2c and Figure 3d. The correlation between PE and *H* is weak for the US market, and thus is not shown here.

One might be intrigued by the relationships between complexity measures (LZ and PE) and the Hurst exponents, as the former quantifiers are estimated for a particular time scale while the latter one considers a time scale range. To understand this monoscale (LZ and PE) versus multiscale (Hurst exponent) approaches, it is best to consider data in terms of patterns. The long-range correlations captured by the Hurst exponent amount to certain special patterns in the data. The patterns in turn determine the values of LZ and PE.

## 4. Discussion

Capital markets sometimes exhibit behaviors that are inconsistent with the EMH. Are deviations from the EMH equally likely to occur with both developed and emerging markets? To gain insights into this question, we compared the behaviors of the US and the Chinese stock markets by computing the LZ complexity and the PE from two stock composite indices, the SSE and the DJIA. We found that the US stock market is largely fully random and consistent with the EMH, irrespective of whether low- or high-frequency stock index data are used. The Chinese stock market is also largely consistent with the EMH when low-frequency data are used. However, a completely different picture emerges when the high-frequency stock index data are used, irrespective of whether the LZ or PE is computed. In particular, the PE decreases substantially in two significant time windows, each encompassing a rapid market rise and then a few gigantic stock crashes. To further clarify the mechanism of complexity changes, we examined the memory effect from the USA and the Chinese minute-to-minute stock index data by computing the Hurst parameter *H* on each day. As expected, *H* is always close to 1/2 for the US stock market. However, in stark contrast, the fractal scaling for the Chinese stock market showed two scaling regimes, one is for time scales up to about 10 minutes, where *H* deviates from 1/2 significantly, the other for time scales from about 10 minutes to a day, where the systematic variations in *H* between 2004 and 2016 suggest three periods, consistent with what the PE indicated.

The significant deviations from the EMH in the Chinese stock market strongly suggests the existence of irrational behavior in the Chinese markets. Indeed, large-scale collective behaviors, such as herding effects [67,68], were frequently observed in the Chinese stock market in 2015 [69]. Large scale manipulation of the market also occurred, as was revealed by the arrest of a number of high-profile investors.

Our analysis strongly suggests that the complexity measures employed here can effectively forewarn of problems in the Chinese market. This is revealed by the wild variations in the LZ complexity and the short time scale Hurst parameter, as well as by the PE and the long-time scale Hurst parameter, which dropped significantly during the strong bull market periods both in the middle of 2006 and at the end of 2014. To make the Chinese and other emerging capital markets more healthy, and to promote more effective cooperation among different economies (which is essential for the successful continuation of globalization), methods for forewarning of deviations from the EMH of a market are invaluable. Thus, by monitoring the complexity changes in the market, it might be possible to guide regulators to the exact mechanisms responsible for the deviations.
